# Developing synthetic tools to decipher the tumor–immune interactome

**DOI:** 10.1073/pnas.2306632120

**Published:** 2023-10-23

**Authors:** Orr-El Weizman, Sophia Luyten, Peiwen Lu, Eric Song, Kai Qin, Darius Mostaghimi, Aaron M. Ring, Akiko Iwasaki

**Affiliations:** ^a^Department of Immunobiology, Yale University School of Medicine, New Haven, CT 06520; ^b^Department of Ophthalmology, Yale University School of Medicine, New Haven, CT 06520; ^c^Department of Molecular Cellular and Developmental Biology, Yale University, New Haven, CT 06520; ^d^HHMI, Chevy Chase, MD 20815

**Keywords:** cancer immunology, immunotherapy, surveillance, T cells, myeloid cells

## Abstract

The ability of immune cells to directly interact with transformed cells is an essential component of immune surveillance and critical for optimal tissue function. The tumor–immune interactome (the collective cellular interactions between oncogenic cells and immune cells) is distinct and varied based on the tissue location and immunogenicity of tumor subtypes. However, comprehensive landscape and the consequences of tumor-interacting immune cells in the tumor microenvironment are not well understood. Current tools are limited in their ability to identify and record interactors in vivo or be utilized for downstream analysis. Here, we describe the development and validation of a technology leveraging synthetic Notch receptors reporting physical tumor cell–immune cell contact in vivo in order to decipher the tumor–immune interactome. We call this approach, Tumor–Immune Interactome Non-biased Discovery Retroviral Reporter or TIINDRR. Using TIINDRR, we identify the tumor–immune interactomes that define immunological refractory and sensitive tumors and how different immunotherapies alter these interactions. Thus, TIINDRR provides a flexible and versatile tool for studying in-vivo tumor–immune cell interactions, aiding in the identification of biologically relevant information needed for the rational design of immune-based therapies.

## Significance

The tumor–immune interactome (the collective cellular interactions between oncogenic cells and immune cells) is distinct and varied based on the tissue location and immunogenicity of tumor subtypes. Since each tissue has a unique immune landscape of circulating and tissue-resident immune cells, the nature of these interactions is dynamic during the formation and persistence of cancer progression. Identifying cellular interactors that surveil the tumor microenvironment in either promoting or antagonizing roles will help identify biologically relevant information that can aid in designing immune-based therapies. In this work, we present a tool that can uncover the tumor–immune interactome. We applied this tool to understand the landscape of interacting immune cells during different tumor subtypes and immunotherapeutic interventions.

Immune cells actively surveil the tissue to detect and correct deviations to maintain homeostasis, by sampling both soluble factors and cell surface proteins (1, 2). Well known in this paradigm is discerning self vs. non-self, by recognition of pathogen-specific molecular patterns by immune cells (3, 4). The nature and the mechanisms by which immunological cell types interact with tumor cells, given that tumor cells are noninfectious and are recognized as self, remain unanswered. Currently, therapeutic targeting of immune cells has revealed the potency of harnessing the immune system in eliminating cancer cells. However, many cancers remain unresponsive to immunotherapies and many patients succumb to eventual metastatic disease. Therefore, understanding the consequences of immune cell interaction with tumor cells is critical to improving response rates to immune therapies.

Previous efforts to define the tumor–immune interactome have utilized several approaches. Different forms of microscopy have revealed where intratumoral immune cells are located relative to the vasculature or tumor cells but are unable to be isolated or utilized for downstream analysis of interacting cells (5–8). Transcriptomic-based approaches, either applying cell-cell proximity assumptions or analyzing known receptor–ligand pairs to infer tumor–immune interactions, are not conducive to direct reporting of confirmed interactors (9, 10). Synthetic tools have been recently developed to uncover cell–cell interactions. For example, tumor-secreted cell-penetrating fluorescent proteins have labeled the surrounding metastatic niche around tumor cells (11). However, these methods report on the cellular compositions of tumor cells’ surrounding niches as opposed to physical tumor-cell interactions. Additionally, intracellular (ic) enzymatic proximity labeling has been used to monitor interacting cells within a defined receptor–ligand pair, such as CD40:CD40L between DCs and CD4^+^ T cells in the tumor-draining lymph node (12, 13). This tool has recently been modified to function outside known receptor–ligand pairs providing valuable insights into the dynamics of immune cell interactions in the gut (14). However, the use of an injectable substrate complicates a primary or metastatic tumor-specific approach to defining immune interactions.

**Fig. 1.**
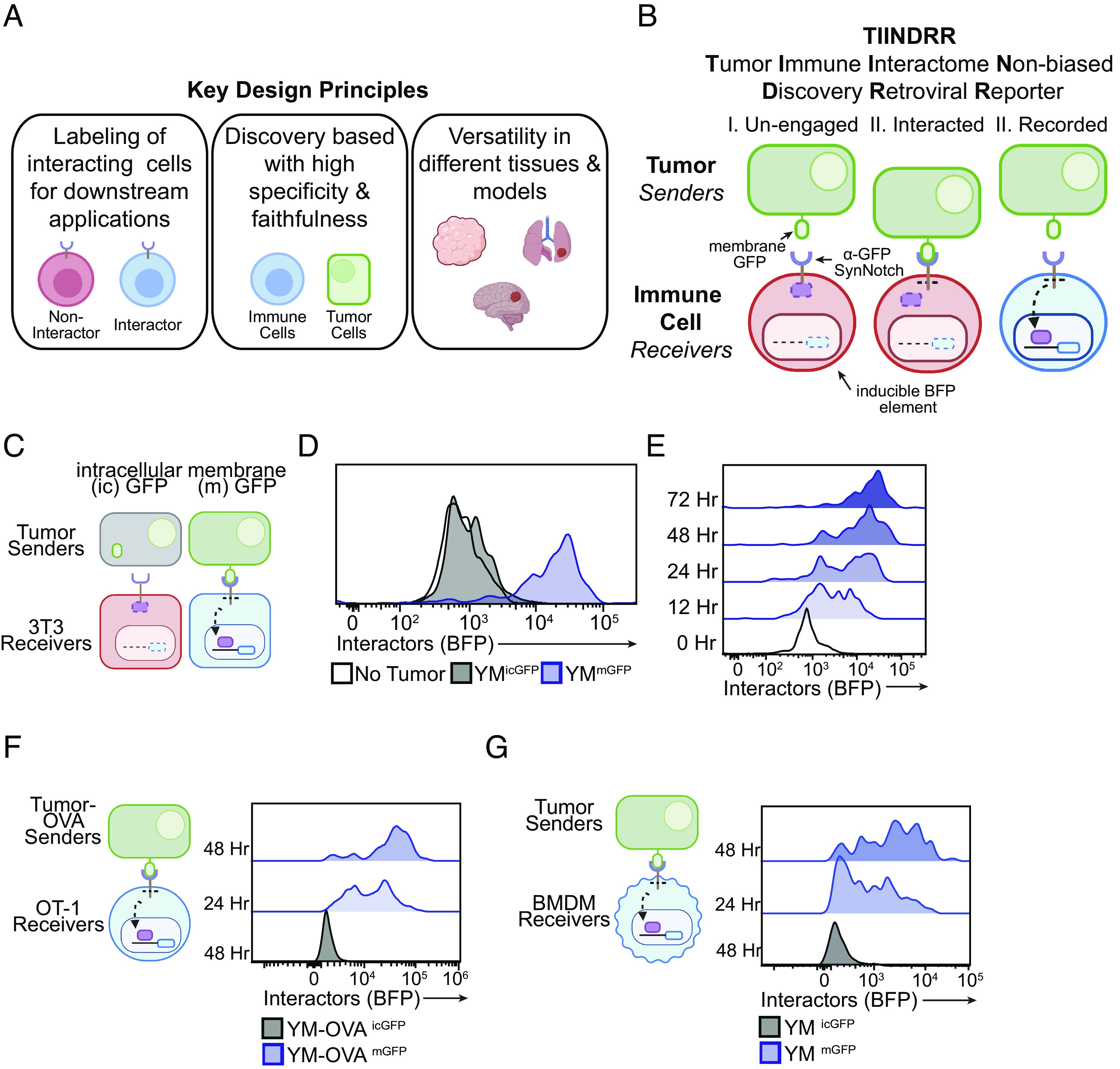
Development of TIINDRR to track tumor–immune interactions. (*A*) Key design principles governing the development of a nonbiased discovery reporter for tumor–immune interactions. (*B*) Schematic representation of TIINDRR approach to tagging immune cells (receivers) interacting with tumors cells (senders). (*C*) Schematic of experiment. 3T3 receivers were cocultured with membrane-bound (m) GFP and ic GFP analyzed by flow cytometry at indicated time points. (*D* and *E*) Representative histogram of 3T3 receiver BFP expression in (*D*) indicated coculture condition and indicated time points. (*F* and *G*) Representative histogram of BFP expression in (*F*) OT-1 CD8^+^ T cell receivers cocultured with YUM^OVA^-mGFP senders and (*G*) BMDM receivers cocultured with YUM-mGFP sender harvested at indicated time points. Data are representative of at least two to three independent experiments with at least n = 3 mice per group.

To better understand the dynamics of the tumor–immune interactome, we set out to develop a genetic-based cell labeling system that achieves three key design principles: i) can label tumor-interacting immune cells for downstream analysis, ii) is discovery-based with a high degree of specificity and faithfulness, and iii) is versatile in different tissues and cancers models (Fig. 1*A*). Here, we leverage previously available genetic and synthetic tools that translate a tumor–immune cell physical contact into recorded read out enabling subsequent monitoring and tracing of the interactome in vivo (15–17). We call this discovery-based cell tagging elucidating physical tumor–immune interactions TIINDRR (Fig. 1*B*). TIINDRR enables the identification of tumor–immune interactions both in vitro and in vivo, uncovering unique interactomes that define distinct tumor subtypes and effects of cancer immunotherapies modalities, providing a valuable tool for cancer-immunology investigations.

## Results

### Development of SynNotch (Synthetic Notch)-Based System for Recording Immune Cell–Tumor Cell Interactions.

Notch is a unique surface receptor where engagement with its cognate extracellular ligand induces the mechanical cleavage of an ic transcriptional regulator domain (18). This can be synthetically adapted to be combined with user-defined extra- and intra-cellular domains (SynNotch) (15). Using validated GFP-detecting nanobody (LaG-17; 50 nM affinity) SynNotch receptor (19–21), we developed a synthetic-based labeling system where tumor “senders” expressing membrane-bound GFP (mGFP) are recognized by immune “receiver” cells expressing an α-GFP SynNotch receptor element (Fig. 1*B*; see *SI Appendix*, Fig. S1*A*). Immune–tumor interactions are recorded when immune receiver cells physically engage with tumor sender cells, resulting in the ic cleavage of the transcriptional activator Gal4, which activates expression blue fluorescent protein (BFP) response element, allowing for the identification and recording of cellular interactions in vivo (Fig. 1*B*, see *SI Appendix*, Fig. S1*A*). Using an artificial ligand (mGFP) and synthetically engineered receptor (anti-GFP SynNotch) with its own signaling domain restricts infringement of cell-specific signaling cascades and allows for a discovery-based approach not limited by known receptor–ligand pairs that define the tumor–immune interactions. Additionally, to expand the cellular versatility of our system, we optimized retroviral dual vector transduction systems to generate immune receivers with both receptor and response elements across several immune cell types (*SI Appendix*, Fig. S1*B*). Thus, we refer to this discovery-based cell tagging system as Tumor–Immune Interactome Non-biased Discovery Retroviral Reporter or TIINDRR.

To test this strategy, we employed syngeneic mouse melanoma YUMM1.7 (YUM) tumor cell line (22) expressing mGFP and cocultured with NIH/3T3 (3T3) adherent cells and analyzed for the induction of BFP (Fig. 1*C*). To establish specificity, 3T3 receivers were also cocultured in vitro with YUM cells expressing ic GFP or without senders. Flow cytometric analysis demonstrated that BFP induction occurred when incubated with YUM-mGFP senders and not in either control setting (Fig. 1*D*). BFP induction reported 3T3-tumor interactions as early as 12 h after coculture and reached maximal BFP expression by 72 h (Fig. 1*E*). Additionally, 3T3 receivers were able to recognize tumor senders with varying degrees of mGFP expression (*SI Appendix*, Fig. S1*C*). To quantify the dynamics of the interaction that TIINDRR reports on, we performed a pulse–chase experiment by sort purifying and CFSE labeling 3T3 receivers following a 24-h coculture with YUM-mGFP senders and monitored BFP expression over time (*SI Appendix*, Fig. S1*C*). BFP expression was maintained for at least 48 h and correlated with dilution of CFSE (*SI Appendix*, Fig. S1 *D*–*F*), indicating that BFP expression denotes an interaction occurring in the prior 48-h window and is not maintained in proliferating daughter cells. We next sought to determine the capabilities of TIINDRR utilizing immune cell receivers in vitro. We generated polyclonal CD8^+^ T cell and OT-1 CD8^+^ T cell receivers and cocultured them with YUM-mGFP senders or YUM^OVA^-mGFP (23) respectively. In both coculture conditions, CD8^+^ T cell receivers increased BFP expression (Fig. 1*F*; see *SI Appendix*, Fig. S1*G*), with faster time to BFP expression for antigen-specific T cells. Thus, TIINDRR can report on both antigen-specific and nonspecific CD8^+^ T cell interactions with tumor cells. Additionally, bone marrow–derived macrophage (BMDM) receivers cocultured YUM-mGFP senders exhibited increased BFP expression (Fig. 1*G*), with similar kinetics to 3T3 receivers. Thus, TIINDRR can report on tumor-CD8^+^ T cell and macrophage interaction in vitro.

### Development and Validation of TIINDRR Recording Immune Cell–Tumor Cell Interactions In Vivo.

**Fig. 2.**
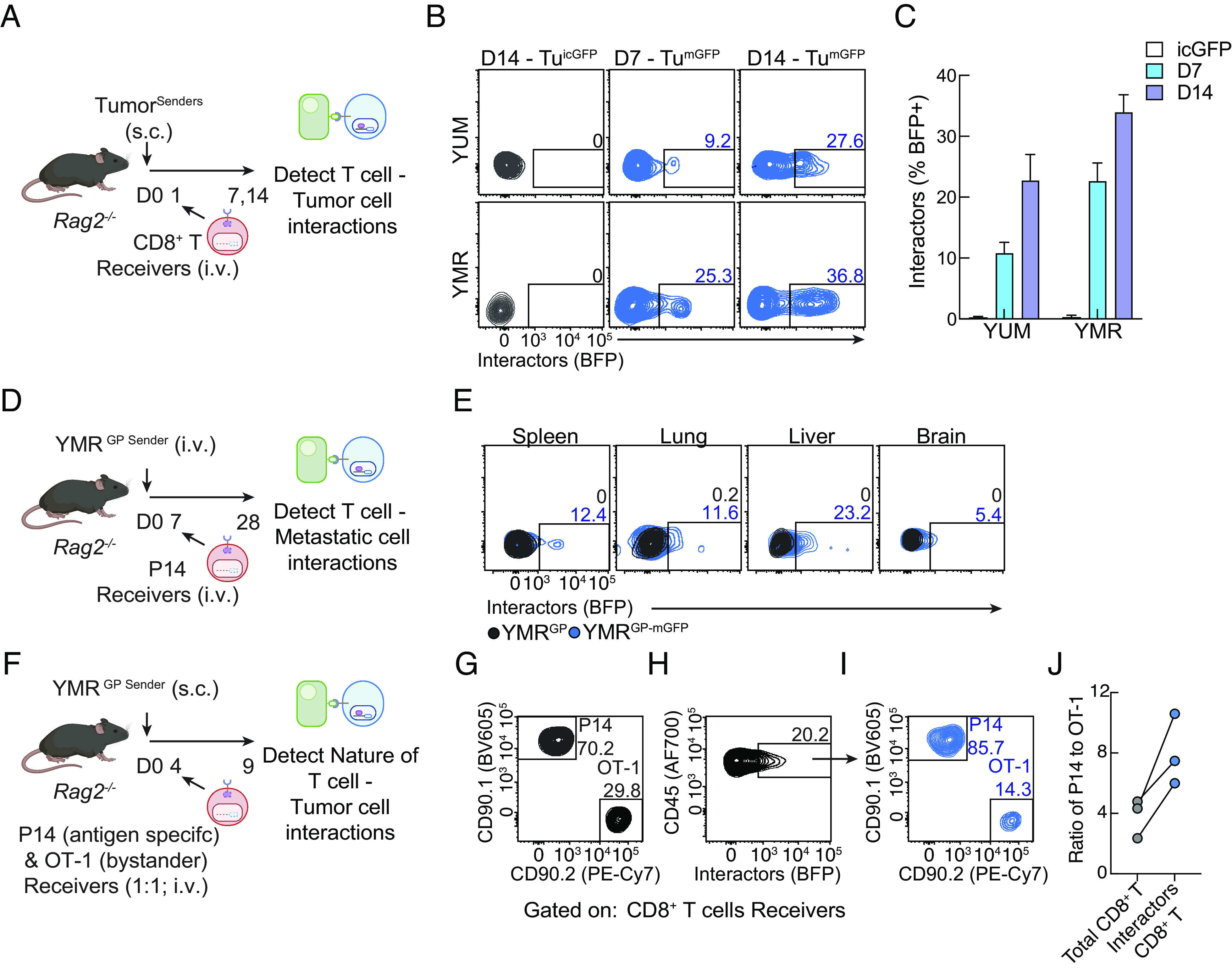
TIINDRR enables recording of CD8^+^ T cells interacting with tumor. (*A*) Schematic of experiment. On day 0, *Rag2*^−/−^ were engrafted with indicated tumor senders s.c., and on day 1, CD8^+^ T cell receivers were adoptively transferred i.v. and recovered from the tumor on day 7 or 14 and assessed by flow cytometry. (*B*) Representative histograms and (*C*) quantification of BFP expression in intratumoral CD8^+^ T cell receivers. (*D*) Schematic of the experiment. *Rag2*^−/−^ mice were engrafted with metastatic YMR^GP^ senders i.v. 7 d later, P14 CD8^+^ T cell receivers were adoptively transferred i.v. and recovered from indicated metastatic disseminated tissues on day 28 and assessed by flow cytometry. (*E*) Representative histograms of BFP expression from harvested CD8^+^ T cell receivers from indicated tissues. (*F*) Schematic of experiment. On day 0, *Rag2*^−/−^ were engrafted with metastatic YMR^GP^ senders s.c. On day 4, equal ratio of OT1 and P14 CD8^+^ T cell receivers were adoptively transferred i.v. and harvested from the tumor on day 9 and assessed by flow cytometry. (*G* and *H*) Representative flow plot of (*G*) P14:OT1 ratio and (*H*) BFP expression of total CD8^+^ T cell receivers. (*I*) Representative flow plot and (*J*) paired quantification of P14:OT1 ratio of interacting BFP^+^ receivers. Data are representative of at least two independent experiments with at least n = 3 mice per group.

To determine whether TIINDRR can function in vivo, we adoptively transferred polyclonal CD8^+^ T cell receivers systemically into *Rag2*^−/−^ mice with established subcutaneous YUM-mGFP senders (Fig. 2*A*). Intratumoral receiver CD8^+^ T cells displayed increased BFP, but not in YUM-icGFP tumors, with expression increasing over the course of tumor progression (Fig. 2 *B* and *C*). Additionally, intratumoral receiver CD8^+^ T cells from YUMMER1.7 (YMR)-mGFP senders, the immunologically sensitive counterpart of YUM, showed enhanced BFP expression (Fig. 2 *B* and *C*); consistent with evidence that YMR is sensitive to CD8^+^ T cells as opposed to YUM (24). Notably, we observed BFP^+^ receivers in the spleen but not the tumor-draining lymph node (*SI Appendix*, Fig. S2 *A* and *B*), indicating that following tumor encounter some interacting CD8^+^ T cells exit to the periphery but do not reengage with the tumor-draining lymph node. To assess the capability of TIINDRR to report tumor–immune interactions in diverse tissues, we adoptively transferred antigen-specific P14 CD8^+^ T cell receivers systemically into *Rag2*^−/−^ mice with established YMR-mGFP metastases expressing the LCMV GP antigen (YMR^GP^) (Fig. 2*D*). Notably, we observed a proportion of interacting CD8^+^ T cells recovered from sites of metastatic dissemination such as the spleen, lung, liver, and brain (Fig. 2*E*). We next assessed whether the introduction of a synthetic high-affinity anti-GFP nanobody with its cognate ligand mGFP could influence tumor–immune interactions. We adoptively transferred P14 CD8^+^ T cells transduced with either the SynNotch receptor element or the inducible BFP element into YMR^GP^-mGFP tumor-bearing mice. We observed no difference in the absolute frequency of tumor-infiltrating CD8^+^ T cells nor an impact on tumor burden (*SI Appendix*, Fig. S2 *C* and *D*), suggesting that TIINDR does not affect accumulation or drive adverse effector function of CD8^+^ T cells in the tumor.

To test the specificity and nature of intratumoral CD8^+^ T cell interactions TIINDRR records, we investigated the ability to report antigen-specific or bystander CD8^+^ T cell responses. We adoptively transferred both antigen-specific P14 and bystander OT-1 CD8^+^ T cells into established YMR^GP^-mGFP subcutaneous tumors (Fig. 2*F*). Although adoptively transferred in equal ratio (50:50; Fig. 2*F*), tumor antigen-specific P14s expanded in the tumor compared to bystander OT-1s (70:30; Fig. 2*G*). However, the ratio of tumor-interacting CD8^+^ T cells is further enriched for P14s over OT-1s (85:15; Fig. 2 *H*–*J*). Thus, suggesting that in addition to an increase in the frequency of P14s, CD8^+^ T cells preferentially interact with the tumor cells in an antigen-specific manner, likely through TCR-MHC surface interactions. Collectively, our results highlight how TIINDRR can be used to track and record various CD8^+^ T cell–tumor interactions both resident to the tumor and in circulation.

To investigate the phenotypic nature of interacting CD8^+^ T cells, we adoptively transferred OT-1 CD8^+^ T cells into established YUM^OVA^-mGFP subcutaneous tumors. We harvested intratumoral OT-1 receivers on days 8 and 13 post tumor injection and performed phenotypic analysis of surface markers using flow cytometry. Interacting OT-1 cells exhibited high coexpression of PD-1^+^Tim3^+^ and CX3CR1^hi^KLRG1^+^, which increased over time (*SI Appendix*, Fig. S2*E*). Additionally, we observed that interacting OT-1 cells expressed higher levels of CD39 compared to noninteracting cells (*SI Appendix*, Fig. S2*E*), consistent with chronic TCR engagement (25, 26). Taken together, our data demonstrate that interacting antigen-specific CD8^+^ T cells are predominantly differentiated into terminal effector cells that are physically engaging with tumor cells.

### Generation of TIINDR Retrogenic Mice.

**Fig. 3.**
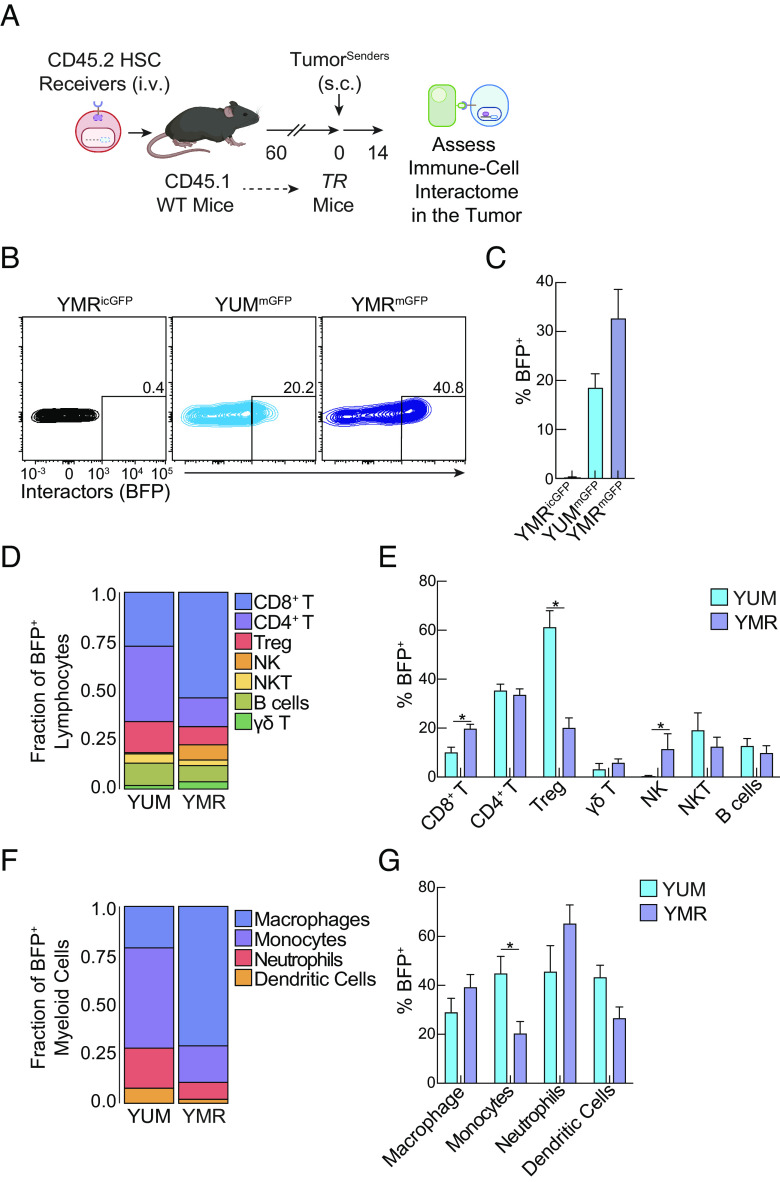
Using TIINDRR to define tumor–immune interactome of immunologically sensitive and refectory tumors. (*A*) Schematic of the experiment. TIINDRR retrogenic mice (TR) were generated and subsequently challenged with either YUM or YMR senders s.c. On day 14 post injection, intratumoral immune cells were analyzed by flow cytometry (see *Materials and Methods* for gating strategy). (*B*) Representative histogram and (*C*) quantification of BFP expression in total CD45.2^+^ receiver expressing cells from indicated conditions. (*D*) Proportion of indicated lymphocyte cell population assessed by absolute cell number of BFP expressing cells. (*E*) Quantification of BFP expression in indicated lymphocyte population. (*F*) Proportion of indicated myeloid cell population assessed by absolute cell number of BFP expressing cells. (*G*) Quantification of BFP expression in indicated myeloid population. Data are representative of at least two independent experiments with at least n = 3 mice per group. The sample is compared using unpaired Student’s *t* test, and data are presented ± SEM. (*P < 0.05).

Based on these initial findings, we generated TIINDRR retrogenic (TR) mice by transducing hematopoietic stem cells (HSCs) with retroviral constructs encoding both BFP response element and SynNotch receptor element and transplanting these HSCs into irradiated congenically distinct hosts (Fig. 3*A*; see *SI Appendix*, Fig. S1*B*). Following reconstitution, the donor hematopoietic compartment are competent receiver cells, thus creating immune receivers across multiple lymphocyte and myeloid lineages. BFP expression was observed in YMR-mGFP, not YMR-icGFP, tumor-bearing TR mice (*SI Appendix*, Fig. S3*A*), indicating that TR mice faithfully report on immune receivers with tumor senders. We tested TR mice ability to record and define two distinct and well-studied tumor–immune interactomes: immunologically refractory YUM-mGFP and immunologically sensitive YMR-mGFP subcutaneous tumors (22, 24) (Fig. 3*A*). Globally, we observed a significant increase in the proportion of immune receivers in contact with tumor senders in YMR-mGFP compared to YUM-mGFP (Fig. 3 *B* and *C*). We were able to identify interactors and noninteractors based on BFP expression by flow cytometry across 8 lymphocyte immune populations (CD8^+^ T cells, CD4^+^ T cells, Tregs, γδ T cells, Natural Killer (NK) cells, Natural Killer T (NKT) Cells, Innate Lymphoid Cells (ILCs), and B cells) and 5 myeloid populations (macrophages, monocytes, neutrophils, and class 1 and 2 dendritic cells) (*SI Appendix*, Fig. S3 *B*–*F*). Analysis of lymphocyte populations revealed that the tumor–immune interactome in YUM-mGFP had a larger proportion of CD4^+^ T cells and Tregs interacting with the tumor compared to YMR-mGFP (Fig. 3*D*), with Tregs having the highest percentage of BFP^+^ expressing cells (Fig. 3*E*). Conversely, the tumor–immune interactome in YMR mGFP had a larger proportion of CD8^+^ T cells and NK cells interacting with the tumor (Fig. 3*D*), with both populations displaying increased BFP expression in YMR compared to their YUM counterparts. Our findings are consistent with YMR tumors supporting an immunologically active tumor milieu that is sensitive to effector lymphocytes (24).

TIINDRR was further able to resolve distinct myeloid interactomes distinguishing between immunologically sensitive and refractory tumor environments. TIINDRR identified that monocytes, neutrophils, and dendritic cells were a significantly increased proportion of the YUM myeloid interactome compared to macrophages making up approximately 75% of immune interactors in the YMR myeloid interactome (Fig. 3*F*). Monocytes were the only population to display significantly higher BFP expression in YUM compared to YMR, with a comparable percentage of BFP interactors in macrophages, neutrophils, and dendritic cells (Fig. 3*G*). Thus, our data collectively demonstrate that TIINDRR is an efficient, specific, and versatile tool to label immune cells and define the tumor–immune interactome in vivo.

### Deciphering the Tumor–Immune Interactome Following Cancer Immunotherapy.

**Fig. 4.**
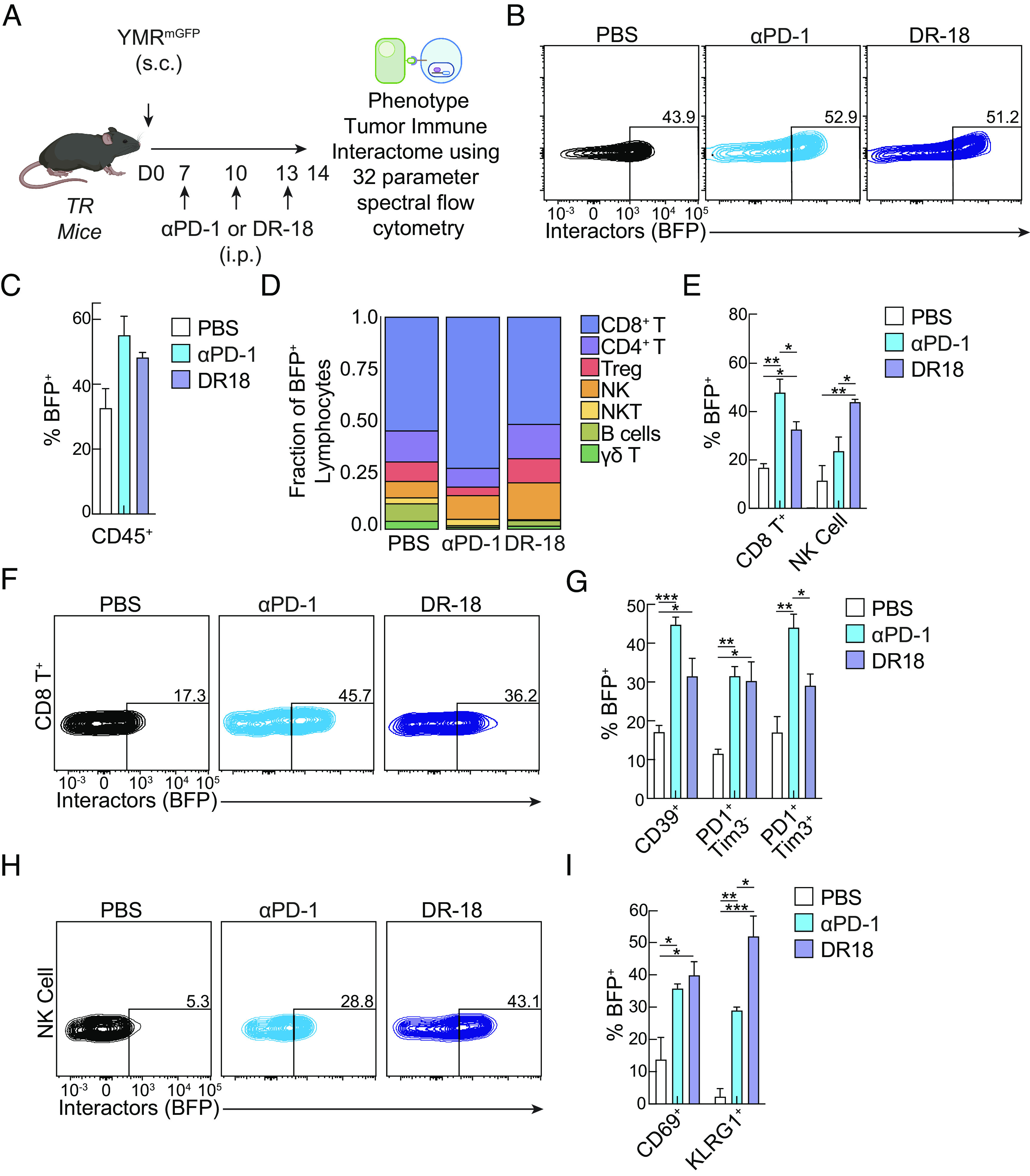
TIINDRR identifies distinct tumor–lymphocyte interactions following cancer immunotherapy. (*A*) Schematic of the experiment. TR mice were generated and subsequently challenged with YMR senders s.c. On days 7, 10, and 13, mice were treated with either vehicle, DR-18, or αPD-1. 14 days post injection, intratumoral immune cells were analyzed by flow cytometry. (*B*) Representative histogram and (*C*) quantification of BFP expression in total CD45.2^+^ receiver expressing cells from indicated conditions. (*D*) Proportion of indicated lymphocyte cell population assessed by absolute cell number of BFP expressing cells. (*E*) Quantification of BFP expression in CD8^+^ T cell and NK cells. (*F*) Representative flow plots of BFP expression in CD8^+.^ T cells. (*G*) Quantification of BFP expression in indicated subset of CD8^+^ T cells. (*H*) Representative flow plots of BFP expression in NK cells. (*I*) Quantification of BFP expression in indicated subset of NK cells. Data are representative of at least two independent experiments with n = 3 to 4 mice per group. The sample is compared using unpaired Student’s *t* test, and data are presented ± SEM. (*P < 0.05, **P < 0.01, and ***P < 0.001).

Recent development in immunotherapies has redefined the therapeutic landscape combating cancer, providing significantly improved patient outcomes (27, 28). Although several mechanisms have been elucidated (29), the wide range of effects of these therapies on the entirety of multiple cellular compartments remains unknown. Thus, elucidating how the tumor–immune interactomes that confer successful responses during different immunotherapies can provide a mechanism of therapeutic targets and uncover novel biology. Since a key feature of TIINDRR is the ability to identify the immune interactome in an unbiased discovery-based approach, we decided to employ TIINDRR to interrogate how different cancer immunotherapies impact the tumor–immune interactions. We chose to compare two distinct therapies: immune checkpoint inhibitor anti (α) PD-1 and cytokine-based immunotherapy DR-18 (an engineered decoy-resistant IL-18 variant) (28). We reasoned that this would not only provide a good test for the faithfulness of TIINDRR due to distinct cellular targets, with αPD-1 selectively targeting PD-1 expression primarily on T cells and DR-18 broadly targeting effector lymphocytes that express IL-18 receptor. To investigate the interactomes and their phenotypic identities, we analyzed immune cells harvested from tumor-bearing mice following treatment with either vehicle, αPD-1 or DR-18, utilizing a 32-parameter spectral flow cytometry surface antibody panel identifying immune populations and phenotypic states (Fig. 4*A*).

TIINDRR revealed a distinct and unique landscape of interacting and noninteracting intratumoral immune cells for αPD-1 and DR-18 (*SI Appendix*, Fig. S4 *A*–*E*), with both treatments sharing a similar increased proportion of total immune cells interacting with the tumor compared to the vehicle control (Fig. 4 *B* and *C*). Analysis of the lymphocyte interactome from both treatments revealed increased tumor interactions of CD8^+^ T cells, NK cells, and to a lesser extent CD4^+^ T cells and Tregs, compared to negligible interactions among γδ T cells, NKT Cells, ILCs, and B cells (Fig. 4 *D* and *E*; see *SI Appendix*, Fig. S4 *B* and *C*). Unique to αPD-1 treatment, there was a predominant frequency and proportion of CD8^+^ T cells interacting with tumor cells compared to vehicle or DR-18 treatment (Fig. 4 *D*–*F*). Phenotypic analysis of T cell surface markers highlighted that both treatments resulted in increased intratumoral CD44^+^CD39^+^ antigen-specific, nonbystander, CD8^+^ and CD4^+^ T cell interactors (Fig. 4*G*, See *SI Appendix*, Fig. S4 *F* and *G*). While both treatments, at this time point, resulted in a similarly increased frequency of interacting stem-like memory (PD-1^+^Tim3^-^) CD8^+^ T cells, only αPD-1 resulted in an increased frequency of terminal effector (PD-1^+^Tim3^+^) CD8^+^ T cells (Fig. 4*G*). In contrast, the lymphocyte interactome following DR-18 treatment was defined by an increased frequency and proportion of both NK cells and CD8^+^ T cells (Fig. 4 *D*–*F* and *H*). NK cell interactors from both treatments adopted an activated state (measured by CD69); whereas DR-18 treatment additionally resulted in mature NK cell interactors (measured by KLRG1) (Fig. 4*I*). The superior role of tumor-interacting NK cells following DR-18 treatment is consistent with a cell-specific requirement of NK cells for DR-18 treatment but not αPD-1 (28).

**Fig. 5.**
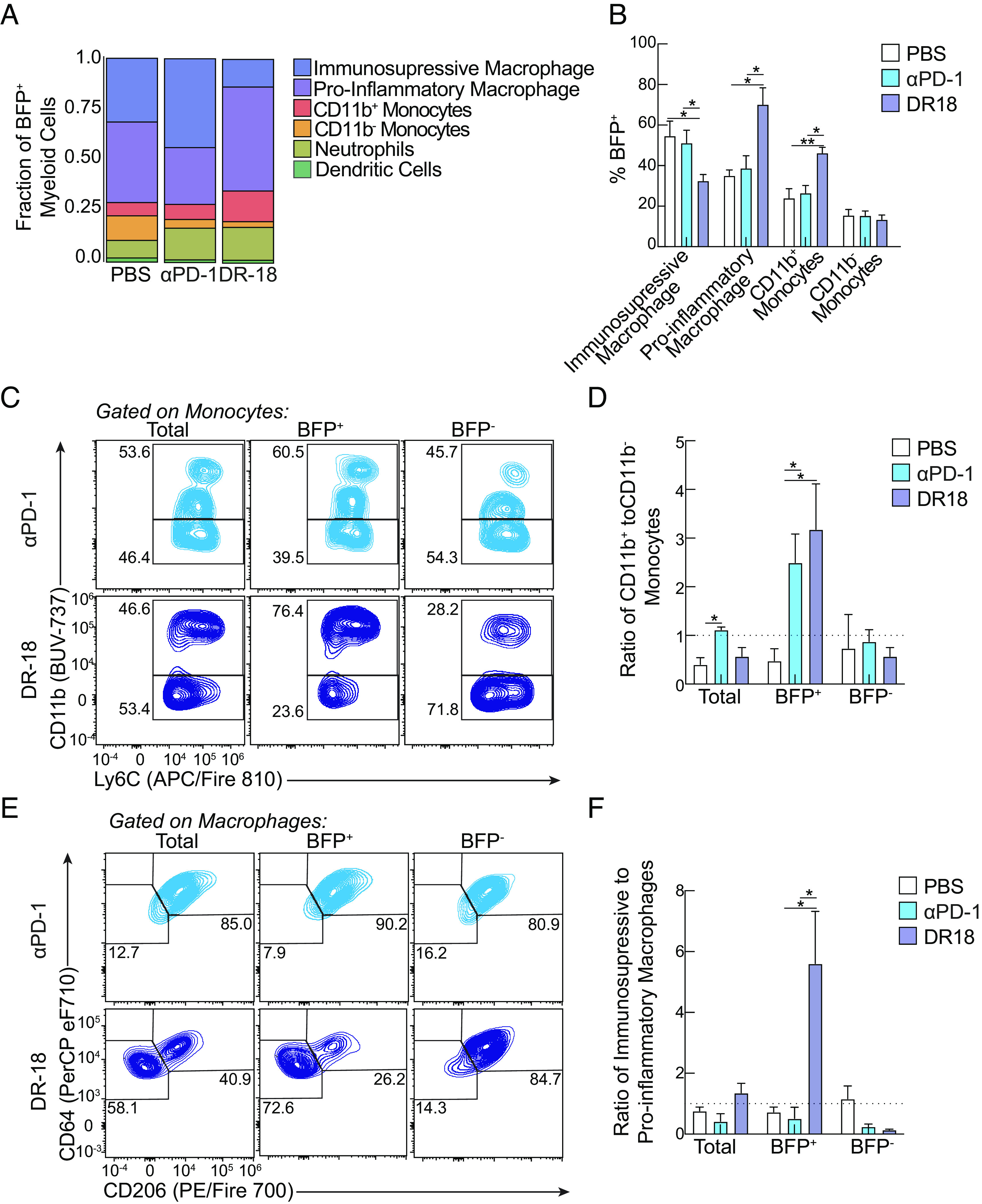
Different modalities of myeloid interactions occur following immunotherapy. TR mice were generated and subsequently challenged with YMR senders s.c. On days 7, 10, and 13, mice were treated with either vehicle, DR-18, or αPD-1. On day 14 post injection, intratumoral myeloid cells were analyzed by flow cytometry. (*A*) Proportion of indicated myeloid cell population assessed by absolute cell number of BFP expressing cells. (*B*) Quantification of BFP expression in indicated myeloid cell population. (*C*) Representative flow plots of CD11b expression on either total, BFP^+^, and BFP^−^ monocytes for each treatment condition. (*D*) Ratio of CD11b^+^ to CD11b^−^ monocytes for each indicated population and treatment condition. (*E*) Representative flow plots of CD206 expression on either total, BFP^+^, and BFP^−^ macrophages for each treatment condition. (*F*) Ratio of proinflammatory to anti-inflammatory macrophages for each indicated population and treatment condition. Data are representative of at least two independent experiments with at least n = 3 mice per group. The sample is compared using unpaired Student’s *t* test, and data are presented ± SEM. (*P < 0.05 and **P < 0.01).

The tumor-myeloid interactome underwent extensive remodeling following both αPD-1 and DR-18 treatments. Like vehicle-treated tumors, most myeloid interactors following either treatment were composed predominantly of macrophages and, to a lesser extent, monocytes, with negligible interactions with dendritic cells (Fig. 5*A*, see *SI Appendix*, Fig. S4 *D* and *E*). Both treatments resulted in an increased fraction of neutrophil interactors [Fig. 5*A*; see *SI Appendix*, Fig. S4 *D*, *E*, and *H*), consistent with reports highlighting that successful immunotherapies require infiltrating neutrophils (30, 31). Compared to vehicle or αPD-1, DR-18 treatment resulted in a unique myeloid interactome enriching for two unique myeloid populations: CD11b+ monocytes and CD206^-^ proinflammatory macrophages, the latter of which made up more than 50% of total DR-18 myeloid interactors (Fig. 5 *A* and *B*). Although αPD-1 treatment did not result in an overall increase in the fraction of total BFP expressing monocytes, both treatments resulted in an enrichment CD11b^+^ over CD11b^−^ monocytes in the interacting portion of monocytes (Fig. 5 *A*–*D*), suggesting tumor-interacting CD11b^+^ monocytes are a hallmark of successful treatment regimen. Unique to DR-18, we observed that proinflammatory macrophages are specifically enriched in the BFP^+^ portion of macrophages compared to BFP^−^ macrophages (Fig. 5 *E* and *F*). While the polarization of proinflammatory macrophages is a feature of DR-18 treatment and in ICI combination of αPD-1 and αCTLA4 (32), our data indicate a specific feature of antitumor proinflammatory macrophages is physical contact with tumor cells. Thus, our data demonstrate the utility of TIINDRR to decode the tumor–immune interactome following perturbations and are a useful tool to uncover immune interactors in the tumor microenvironment.

## Discussion

We introduce TIINDRR, an application of SynNotch receptors for labeling immune cell interactions in the tumor microenvironment, with several useful and adaptable features. The use of a non-host-expressed ligand (mGFP) and synthetic engineered α-GFP SynNotch allows for a discovery-based approach outside of known receptor–ligand pairs. Several reports have expanded the SynNotch toolbox (33–35). Incorporation of these elements could allow for the next generation of TIINDR to recognize multiple senders or combinatorial signals, highlighting the system versatility and flexibility to address specific questions. Since TIINDRR can be genetically encoded there are multiple avenues to create both receivers and senders. In this iteration of TIINDRR, pairing with retroviral transduction allowed for interrogation of several different immune populations. Recently, a Cre-induced system SynNotch receptor transgenic mouse was developed providing an additional source of immune receivers with the capacity to trace transient interactions (36). Similarly, tumor senders can be made utilizing transgenic mice with Cre-inducible membrane targeted ligands, thus creating tumor senders in a variety of GEMM models. Finally, TIINDRR is not limited by the delivery requirements of a labeling substrate, offering the ability to record interactions in vivo in tissues with unconventional vasculature limiting the effectiveness of substrates, such as the brain and the tumor. Even with these advances, there are some limitations to our TIINDRR approaches. Although TIINDRR offers a genetic and synthetic tool to uncover the tumor–immune interactome, the extent to which the affinity of the SynNotch nanobody drives artificial interaction warrants future investigation. Further, the current version of TIINDRR records all possible immune interactions with the senders. Future versions can incorporate an on/off switch to the recording to capture the time course of interactions, as well as pulse–chase tracing of interactomes over time.

Importantly, this approach allows us to generate several avenues of investigation and hypotheses interrogating the tumor–immune interactome. Our adoptive transfer models of CD8^+^ T cells revealed that both bystander and antigen CD8^+^ T cells interact with tumor cells, indicating that TIINDRR reports on a wide range of CD8^+^ T cell sampling of tumor cells. Additionally, we observed that following tumor encounter, some interacting CD8^+^ T cells exit to the periphery but do not reenter the tumor-draining lymph node. Although several reports demonstrate that tumor-specific CD8^+^ T cells can be found in circulation or lymph in patients (37–39), our data suggest that an overwhelming majority of interacting CD8^+^ T cells remain in the tumor, with a minority found in peripheral circulation but not in the tumor-draining lymph node. Additionally, TIINDRR can robustly report interactions across several tissues. Using TIINDR, we were able to immunophenotype tumor–immune interactors. We observed that terminal effector CD8^+^ T cells and activated mature NK cells preferentially interact with the tumor cells. These effector lymphocytes are critical in host antitumor immunity since they have the capacity to target and eliminate tumor cells. Furthermore, we observed that immunosuppressive CD206^+^ macrophages predominate interactions with tumor cells. However, following cytokine-based immunotherapy, these macrophages, that are in physical contact with the tumor, adopted a proinflammatory CD206^−^ phenotype. Our examination of immunologically sensitive and refractory tumor–immune interactome and how immunotherapies modulate these interactions were consistent with several prior reports, confirming the reliability of TIINDRR.

One possible interpretation of TIINDRR-reported tumor–immune interactome is that the increase in tumor-interacting cells is a result of increased immune infiltration into the tumor. Of note, we did not observe differences in CD8^+^ T cell infiltration in the tumor due to the introduction of the SynNotch receptor. Although limited to the cell types tested, our data and other work utilizing SynNotch receptors suggest that TIINDR does not drive or affect immune infiltration into the tumor (15, 19, 36). Whether SynNotch interactions influence alterations to native surface ligand/receptor interactions needs to be assessed on a case-by-case basis. However, an increase in the biologically relevant infiltration of immune cells can result in an increased proportion of interacting immune cells. For example, our comparison of the tumor–immune interactome in immunologically cold (YUM) and hot (YMR) tumors highlights an increase in the proportion of BFP^+^ interacting CD8^+^ T cells in YMR tumors. This is likely a result of increased neoantigens present in YMR tumors that prime and expand CD8^+^ T cells in the draining lymph node (24), increasing the frequency and probability of tumor-interacting CD8^+^ T cells. Conversely, while YMR tumors expanded the absolute frequency of Tregs, we observed a higher proportion of tumor-interacting Tregs in YUM tumors. Additionally, analysis of cognate CD8^+^ T cells demonstrated that the percentage of interacting antigen-specific CD8^+^ T cells was greater than the frequency of tumor-infiltrating cells. Thus, in addition to tumor infiltration and expansion, there are likely immune cell-intrinsic mechanisms that explain the nature of immune interactions in the tumor microenvironment.

TIINDR was able to expand our understanding of existing therapies by identifying previously underappreciated characteristics of tumor-interacting immune cells, such as proinflammatory macrophages following cytokine-based immunotherapy. Collectively, these tumor–immune interactomes provide valuable insight into the nature of immune surveillance and highlight therapeutic targets. Importantly, our results provide a rationale for the combination treatment of αPD-1 and DR-18, each targeting distinct but complementary immune cell types. Future investigations, pairing TIINDRR with transcriptomic analysis, microscopy, and other cell tagging tools will allow for a more comprehensive atlas of the tumor–immune interactome, providing insight into gene regulation governing immune interaction and spatial localization of tumor-interacting immune cells. Thus, TIINDRR provides a useful unbiased tool to analyze the tumor interactome, that can uncover biology to be leveraged for the next generation of cancer immunotherapies.

## Materials and Methods

### Mice.

Mice were bred and maintained at Yale University in accordance with the guidelines of the Institutional Animal Care and Use Committee (IACUC). The following mouse strains were used in this study: C57BL/6J (CD45.2, WT), B6.SJL (CD45.1, WT), *Rag2^−/−^,* CD90.1 P14 TCR transgenic (from N. Joshi), and CD45.1 OT-1 TCR transgenic (from N. Joshi). Unless otherwise specific, all mice were purchased from Jackson Labs. Experiments were conducted using age- and gender-matched mice in accordance with approved institutional protocols.

### Cell Lines.

All tumor cell lines were cultured in DMEM/F12 (ThermoFisher) including L-glutamine and 2.438 g/L sodium bicarbonate and supplemented with 1× nonessential amino acids, penicillin and streptomycin, and l-glutamine (ThermoFisher), and 15% fetal bovine serum (Sigma-Aldrich). NIH/3T3 cell line was cultured in DMEM (ThermoFisher) supplement with 1x penicillin and streptomycin, and l-glutamine (ThermoFisher), and 10% fetal bovine serum (Sigma-Aldrich). All cells were cultured at 37 °C, 5% CO2, and kept at low passage and tested negative for *mycoplasma* contamination. YUMM1.7- OVA was kept under G418 (InvivoGen) selection (Gift from P. Ho) (23). YUMMER1.7 retrovirally transduced with LCMV GP expressing plasmid as previously described (40). LCMV-*gp* was cloned from pHCMV-LCMV-Arm53b; Addgene#15769 and inserted into MSCV-IRES-Thy1.1; Addgene#17442) and sorted for CD90.1 as a selection marker. YUMMER1.7-GP clones was validated in-vivo, by testing the ability to expand transgenic P14 CD8^+^ T cells.

### Generation of Tumor Senders.

YUMM1.7, YUMMER1.7, YUMM1.7-OVA, and YUMMER1.7-GP stably expressing mGFP were generated using lentiviral transduction (Lenti-X, Takara) of pHR-EGFPligand plasmid (Addgene#79129) and sorted for surface GFP expression (A647 α-GFP; BioLegend). All transduced cell lines were selected for single cell clones and matched to parental lines for exhibited similar morphology, in vitro growth characteristics, and in vivo tumor formation.

### Generation of Receivers.

α-GFP SynNotch receptor element and UAS-inducible BFP response element were cloned into retroviral MP71 vector (gift from Grassman S.) using Infusion (Takara). For retrovirus production, a stable retroviral-producing Platinum-E cell line (Cell Bio Labs) was produced with retrovirus form Platinum-A packaging cell line (Cell Bio Labs) transfected with vectors using lipofectamine (Invitrogen). Stable retroviral-producing Platinum-E cell lines for either receptor or response element were selected by FACS (ARIA II) using α-cMyc (BioLegend) and mCherry, respectively. Cell lines were selected with Blasticidin (InvivoGen) and Puromycin (InvivoGen). Fresh retroviral supernatant was used for all experiments and collected 48 to 72 h after passaging and purified from remaining cells by centrifugation at 500 g for at 4 °C for 8 min.

Retroviral transduction of NIH/3T3 receivers was performed by incubating NIH 3T3 with retroviral supernatant of both receptor and response elements at equal ratio for 24 h and sorted for double positive expression of cMyc and mCherry by FACS 72 to 96 h. post transduction.

Retroviral transduction of CD8^+^ T cell receivers was performed by isolating CD8^+^ T cells from spleens of 8- to 20-wk-old donor mice (CD45.1 WT, P14, or OT-1) using the bead-based negative enrichment kit (Stemcell). Cells were incubated at 1*10^6^ cells/mL overnight at 37 °C in cRPMI supplemented with 4 ng/mL hIL-2 (PeproTech) and 2*10^4^/mL CD3CD28 Dynabeads. Retroviral transduction was achieved by spinoculation, where 1 mL of each receptor and response element viral supernatant was loaded onto 6-well untreated tissue culture with RetroNectin (Takara) according to the manufacturer’s instructions at 3,000 g at 32 °C for 2 h. Afterward, 1 mL was discarded, and 1 mL of cells was added in 2x IL-2 supplemented medium in a final concentration of approximately 1 to 2*10^6^ cells per well. Cells were then spinoculated at 800 g at 32 °C for 1.5 h. After 2 d in culture, the transduced T cells underwent AF647 positive selection for AF647 stained cMyc expression (Miltenyi) and subsequently sorted for double-positive expression of both receptor and response element. Following sorting, cells were recovered for 2 h in IL-2 supplemented medium in the incubator prior to use.

Generation of BMDM receivers was performed by harvesting the bone marrow of 8- to 20-wk-old donor mice (CD45.2 WT) and spinoculated on days 1 and 2 postharvest with retroviral supernatant mixed with 4 μL of Lipofectamine per 1 mL of viral supernatant. Cells were maintained at 4 × 10^6^ cells per nontreated tissue culture 10 cm in cRPMI supplement with 30% MCSF supernatant (gift from R. Medzhitov). On day 4, 10 mL fresh media were added. On day 7, cells were replated in supplemented cRPMI at 4 × 10^6 cells per 10 cm plate, and transduction efficiency was tested. Cells were sorted on day 9 for double-positive expression of both receptor and response element.

Generation of TIINDRR retrogenic mice was performed by isolating HSCs from the bone marrow of 8- to 20-wk-old donor mice (CD45.2 WT) using the bead-based negative enrichment kit (Stemcell) and subsequently sorted for Sca-1-positive lineage (CD19, CD3, NK1.1, Gr-1, F4/80) negative expression. Cells were incubated at 37 °C in cDMEM, supplemented with 20 ng/mL mIL-3, 50 ng/mL mIL-6, and 50 ng/mL mSCF (BioLegend), for 3 to 4 d in a tissue-culture-treated 48-well plate at 3 to 4*10^6^/mL. Retroviral transduction was achieved by spinoculation, where 0.2 mL of each receptor and response element viral supernatant was loaded onto 48-well untreated tissue culture with RetroNectin (Takara) at 3,000 g at 32 °C for 2 h. Afterward, 0.2 mL was discarded, and 0.2 mL of cells was added in 2x supplemented medium in a final concentration of approximately 0.4*10^6^ cells per well. Cells were then spinoculated at 800 g at 32 °C for 1.5 h. After 2 d in culture, the transduced HSCs underwent AF647 positive selection for AF647 stained cMyc expression (Miltenyi) and subsequently sorted for double-positive expression of both receptor and response element to at least 90% purity. Following sorting, cells were recovered for 2 h in supplemented medium in the incubator. Cells were suspended in PBS at 0.5*10^6^ cells per 100 µL and injected intravenously (i.v.) into irradiated CD45.1 recipient mice (two times 4.5 Gy, with a resting period of 4 h). Eight weeks post reconstitution in TR mice, the percentage of CD45^+^ cells in the blood and spleen stably expressing TIINDRR constructs ranges from 5 to 20% with an average of ~12%. For experiments, we matched mice with similar immune reconstitution and used mice 8 to 10 wk post reconstitution.

### Coculture Experiments.

For coculture experiments, tumor sender cells were plated at 1*10^3^ cell in a 96 round bottom plate. Twenty-four hours later, following tumor cell proliferation, receivers were plated with the following conditions: NIH/3T3 receivers at 2*10^3^/well, BMDM receivers at 5*10^3^ cells/well, and CD8^+^ T cells 15*10^3^/ well. Following 1 min spin at 250 g, cells were incubated in triplicate for indicated time points and harvested and analyzed for BFP expression by flow cytometry. Experiments using CFSE labeling (ThermoFisher) were performed according to the manufacturer’s instructions.

### Mouse Tumor Studies.

1*10^6^ tumor cells were engrafted subcutaneously (s.c.), or 1*10^5^ tumor cells were engrafted i.v. into *Rag2^−/−^*mice. 2.5*10^6^ tumor cells were engrafted s.c. into TIINDRR retrogenic mice. For all studies, tumor growth was measured twice weekly by caliper (tumor volume = 0.5xlengthxwidth^2) and when appropriate groups were designated to ensure equal average size. For adoptive transfer studies, T cells were resuspended in PBS 1*10^6^ cells per 100 µL and injected i.v. into recipient mice. For treatment studies, treatment was initiated when the mean tumor size was between 50 and 100 mm^3^ (on day 7). Mice were treated with DR-18 at 0.32 mg/kg or αPD-1 at 8 mg/kg by intraperitoneal injection on days 7, 10, and 13 post tumor engraftment. When necessary, mice were killed when tumors reached IACUC established end points (volume greater than or equal to 1,000 mm^3^).

### Isolation of Immune Cells.

Spleens and tumor-draining lymph nodes were physically dissociated using a plunger and 70-µ filter. To isolate cells from the bone marrow, the femur and tibia were harvested and physically dissociated using a pestle and mortar. To isolate lymphocytes from the liver, the tissues were physically dissociated using a glass tissue homogenizer and purified using a discontinuous gradient of 40% over 60% Percoll. Immune cells form the brain, subcutaneous tumors, and lungs were physically disassociated and incubated for 30 min in digest solution [1 mg/mL type D collagenase (Roche) and 10 U/mL DNAseI (Roche) and in RPMI supplemented with 5% fetal calf serum, 1% L-glutamine, 1% penicillin–streptomycin, and 10 mM HEPES]. Immune cells from tumors and brain were further purified using a 50% and 25% Percoll solution respectively. The resulting dissociated tissue was passed through 70-µm strainers and centrifuged. Red blood cells were lysed using ACK lysis buffer.

### Immunophenotyping of Mice.

Cell surface staining of single-cell suspensions from spleens and tumors was performed using fluorophore-conjugated antibodies (BD Biosciences, eBioscience, and BioLegend). All samples were stained with Zombie Yellow or Zombie NIR (BioLegend) to discriminate dead cells and incubated with Fc receptor blocking antibody (Fisher Scientific) and Monoblock (BioLegend) Cell staining was performed using the following fluorophore-conjugated antibodies: I-A/I-E (M5/114.15.2), B220 (RA3-6B2), CD8α (53–6.7), CD4 (GK1.5), TCRβ (H57-597), CD3e (17A2), CD45 (30F11), CD45.1 (A20), CD45.2 (104), Ly6G (1A8), CD44 (IM7), CD19 (6D5), NK1.1 (PK136), KLRG1 (2F1), CD69 (H1.2F3), CD11b (M1/70), CD39 (24DMS1), XCR1 (ZET), PD-1 (29F.1A12), Tim3 (RMT3-23), CD200R1 (OX-110), CD64 (X54-5/7.1), CD11c (N418), Ly6C (HK1.4), cMyc (9E10), GFP (FM264G), CD25 (PC61), CD127 (A7R34), CD206 (C068C2), TCRγδ (UC7-13D5), CD90.2 (53-2.1), and CD90.1 (OX-7). Flow Sorting was performed using ARIAII (BD). Fluorescence spectra were acquired using an Attune NXT (ThermoFisher) or Aurora (Cytek) and analyzed by FlowJo (Version 10). For control staining, naive mice splenocytes or fluorescent minus one (FMO) staining was used.

All cells were gated for Singlets^+^, CD45^+^, and Live Dead^−^. For lymphocytes the following strategy was used, following exclusion of myeloid-specific markers: CD8^+^ T Cell = CD8^+^, CD4^−^, CD90^+^, CD3^+^, TCRαβ^+^, NK1.1^−^, CD19^−^; CD4^+^ T Cell = CD4^+^, CD25^−^, CD8^−^, CD3^+^, TCRαβ^+^, NK1.1^−^, CD19^−^; Treg = CD4^+^, CD25^+^, CD8^−^, CD3^+^, TCRαβ^+^, NK1.1^−^, CD19^−^; γδ T cells = TCRγδ^+^, CD90^+^, CD3^+^, TCRαβ^−^, NK1.1^−^; NK Cell = NK1.1^+^, CD200R1^−^, CD3^−^, CD19^−^; NKT Cell = NK1.1^+^, CD3^+^, TCRαβ^+^, CD19^−^; ILC1 = NK1.1^+^, CD200R1^+^, CD3^−^, CD19^−^; ILC2 = CD200R1^+^, CD127^+^; KLRG1^+^; CD90^+^, NK1.1^−^, CD3^−^, CD19^−^; ILC3 = CD200R1^+^, CD127^+^; KLRG1^−^; CD90^+^, NK1.1^−^, CD3^−^, CD19^−^; B cells = CD19^+^, CD3^−^, TCRαβ^+^, NK1.1^−^. For myeloid cells, the following strategy was used following exclusion of lymphocyte-specific markers: DC1 = CD64^−^, MHCII^+^, CD11c^+^, CD11b^−^, XCR1^+^; DC2 = CD64^−^, MHCII^+^, CD11c^+^, CD11b^+^, XCR1^−^; Macrophages = CD64^+^, CD11b^+^, Ly6G^−^; Monocytes = Ly6C^+^, CD64^−^, Ly6G^−^; Neutrophiles = Ly6G^+^, CD11b^+^, Ly6C^−^, CD64^−^.

### Statistical Analysis.

Statistical analyses were conducted with Prism 8 (GraphPad Software). Ordinary one-way or two-way ANOVA with Tukey’s multiple comparisons test or a two-tailed paired or unpaired Student’s *t* test (labeled in the figure legend) was used to determine statistical significance (*P < 0.05, **P < 0.01, ***P < 0.001, and ****P < 0.0001). The mean and SEM are presented in the figures. The error bars represent the SEM.

## Supplementary Material

Appendix 01 (PDF)

## Data Availability

All study data are included in the article and/or *SI Appendix*.
