# Supplementary material for: Developing synthetic tools to decipher the tumor–immune interactome
Source: Proc Natl Acad Sci U S A. 2023 Oct 23;120(44):e2306632120. doi: 10.1073/pnas.2306632120 (PMC10622925; doi:10.1073/pnas.2306632120)
Supplement: Supplementary file 1 — Appendix 01 (PDF) [file pnas.2306632120.sapp.pdf]

Supporting Information for

Developing synthetic tools to decipher the tumor–immune interactome

Orr-El Weizman[1], Sophia Luyten[1], Peiwen Lu[1], Eric Song[1,3], Kai Qin[1], Darius Mostaghimi[1], Aaron M. Ring[1], & Akiko Iwasaki[1,2,4]

[1]Department of Immunobiology, Yale University School of Medicine, New Haven, CT
[2]Department of Molecular Cellular and Developmental Biology, Yale University, New Haven, CT
[3]Department of Ophthalmology, Yale University School of Medicine, New Haven, CT
[4]Howard Hughes Medical Institute, Chevy Chase, MD

Correspondence

Akiko Iwasaki, PhD
akiko.iwasaki@yale.edu

This PDF file includes:

Figures S1 to S4

Figure S1

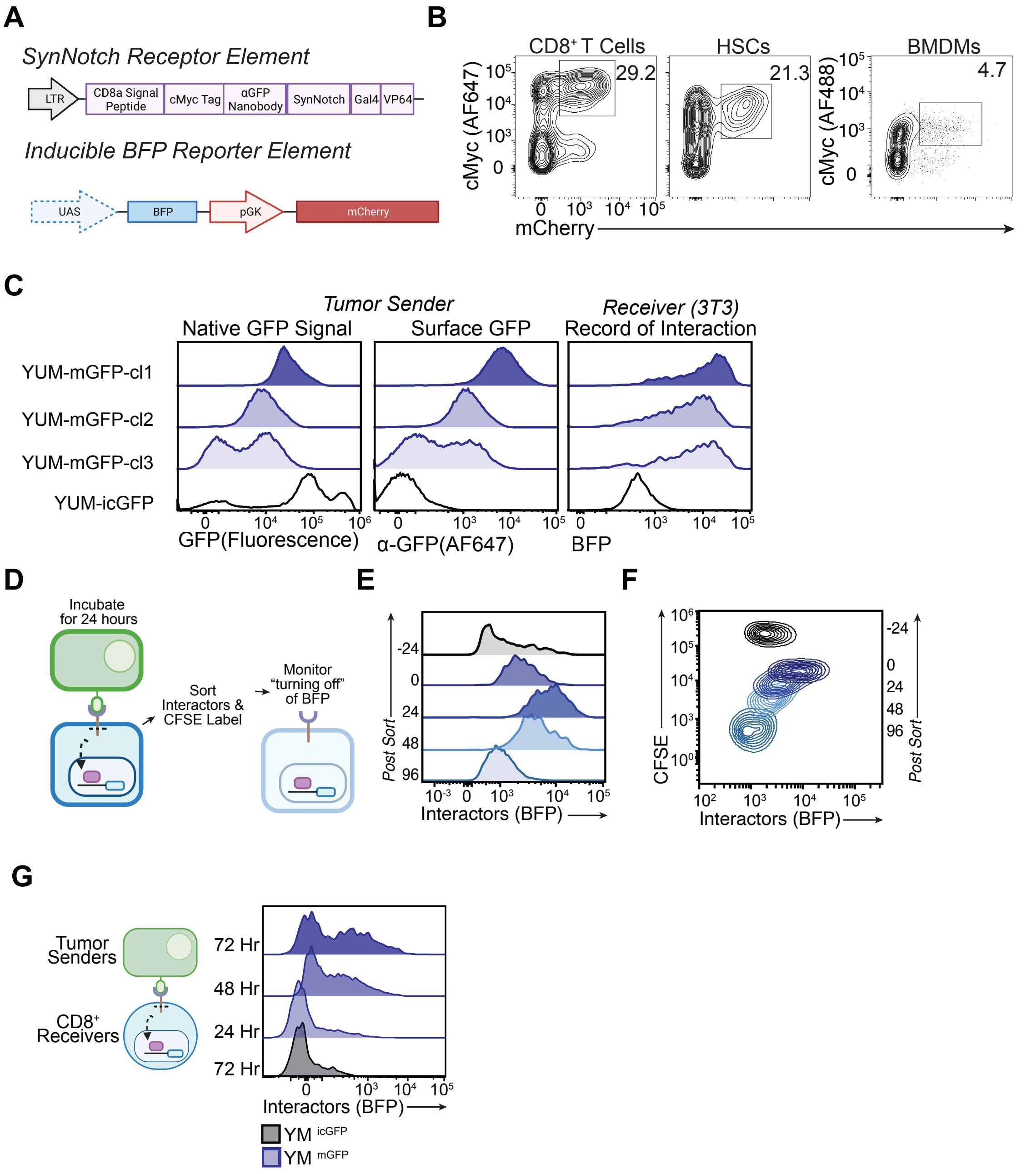

Figure S1. Related to Figure 1. Characteristics of TIINDRR.

(a) Schematic of plasmid components of receptor and response element of TINDRR. **(b)** Retroviral transduction efficiency in CD8$^+$ T cells, Hemopoietic stem cells (HSCs), and bone marrow derived macrophages (BMDMs). **(c)** Senders of varying surface GFP expression and 3T3 receivers were co-cultured and analyzed for BFP. Representative histograms of GFP fluorescence (*left panel*) and surface GFP expression (middle panel) of indicated senders. BFP expression in 3T3 receivers following 72-hour co-culture with indicated senders (right panel). **(d)** Schematic of experiment. 3T3 receivers and tumor senders were co-cultured with mGFP senders. BFP$^+$ 3T3 receivers were

sorted and CFSE labeled. Dilution of proliferative CFSE dye and loss of BFP expression were analyzed. **(e,f)** Representative histogram of BFP expression **(e)** and representative flow plots of CFSE expression **(f)** in 3T3 receivers at indicated time points post sort. **(g)** Representative histogram of BFP expression in polyclonal CD8$^+$ T cell receivers cocultured with YUM-mGFP senders harvested at indicated time points. Data is representative of at least two independent experiments with at least n=3 mice per group.

Figure S2

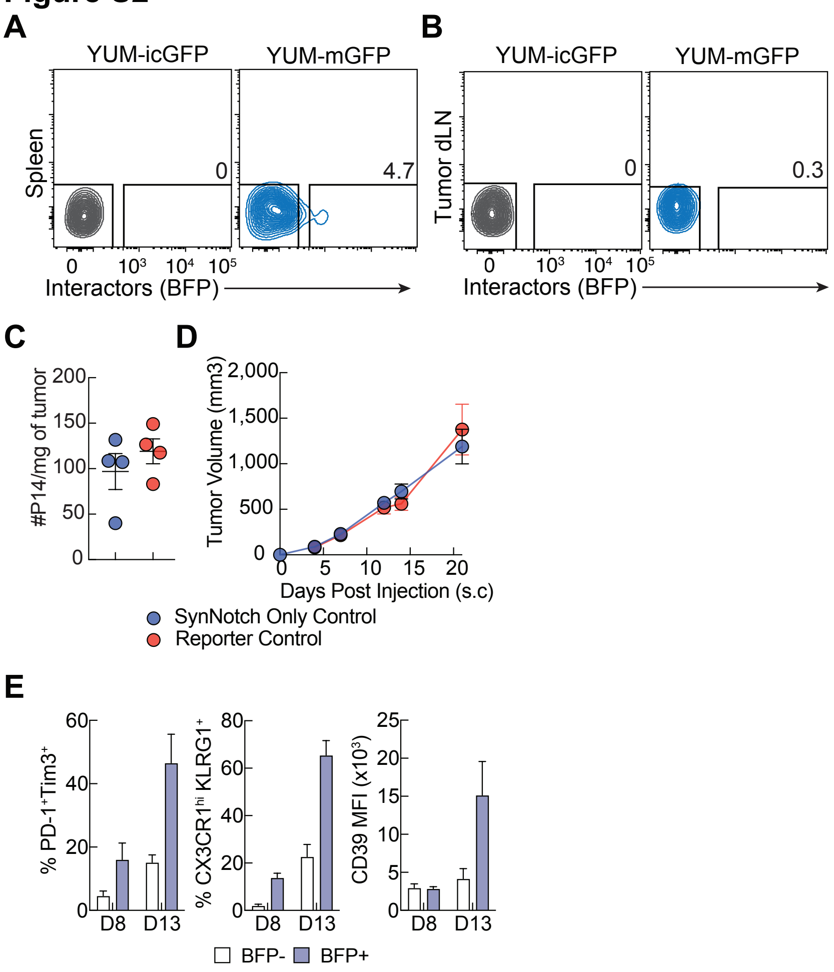

Figure S2. Related to Figure 2. Analysis of interacting T cells using TIINDRR

(a,b) On day 0, *Rag2*$^{-/-}$ mice were engrafted with indicated tumor senders subcutaneously (s.c.) and on day 1 CD8^{+} T cells receivers were adoptively transferred intravenously (i.v.). and recovered from the tumor on day 7 or 14 and assessed by flow cytometry. Representative histograms of BFP expression in harvested CD8^{+} T cells receivers from **(a)** spleen and **(b)** tumor draining lymph node. **(c,d)** On day 0, *Rag2*$^{-/-}$ mice were engrafted with YMR^{GP}-mGFP senders s.c. On day 4, 1*10^{6} P14 CD8^{+} T cells receivers transduced with either only SynNotch receptor element or BFP response element were adoptively transferred i.v. **(c)** Quantification of absolute cell counts of indicated tumor infiltrating P14 cells on day 10 post tumor engraftment. **(d)** Mean YMR^{GP}-mGFP tumor growth in *Rag2*$^{-/-}$ mice with indicated transferred P14 population. **(e)** On day 0, *Rag2*$^{-/-}$ mice were engrafted with indicated YUM^{OVA}-mGFP senders s.c. and on day 3 OT-1 CD8^{+} T cells receivers were adoptively transferred i.v. and recovered from the tumor on day 8 or 13 and assessed by flow cytometry for indicated cell surface marker expression. Data is representative of at least two independent experiments with at least n=3 mice per group.

Figure S3

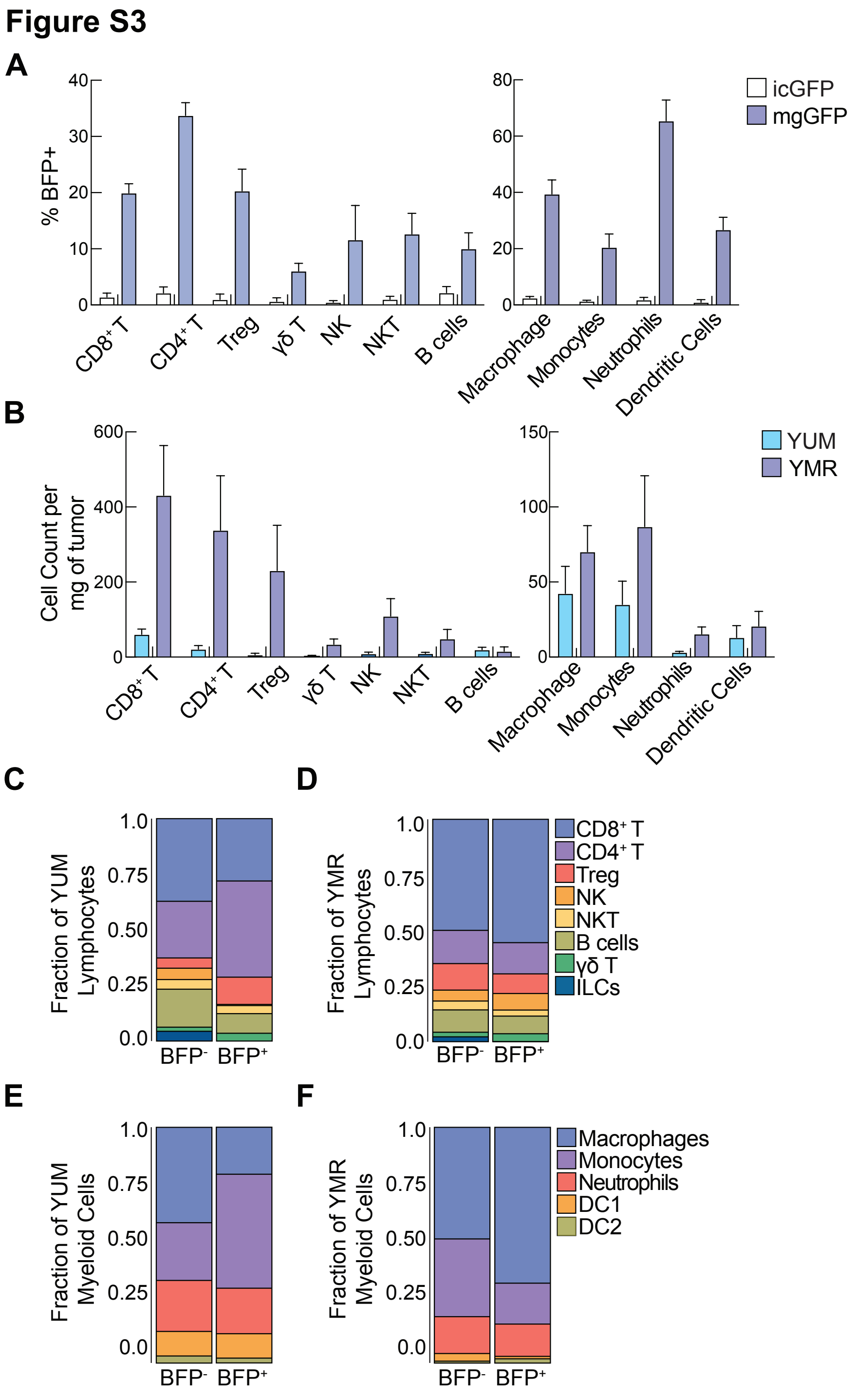

Figure S3. Related to Figure 3. Tumor-Immune interactomes in immunological refractory and sensitive tumors.

TR were generated and subsequently challenged with either YUM or YMR senders s.c. On day 14 post injection, intratumoral immune cells were analyzed by flow cytometry. **(a)** Quantification of BFP expression in indicated immune cell population harvest from YMR-icGFP and YMR-mGFP tumors. **(b)** Quantification of absolute cell counts in indicated immune cell populations in YUM-

mGFP and YMR-mGFP tumors. **(c,d)** Proportion of indicated lymphocytes cell population in YUM **(c)** and YMR **(d)** tumor models assessed by absolute cell number of BFP^+ or BFP^- cells. **(e,f)** Proportion of indicated myeloid cell population in YUM **(e)** and YMR **(f)** tumor models assessed by absolute cell number of BFP^+ or BFP^- cells. Data is representative of at least two independent experiments with at least n=3 mice per group.

Figure S4

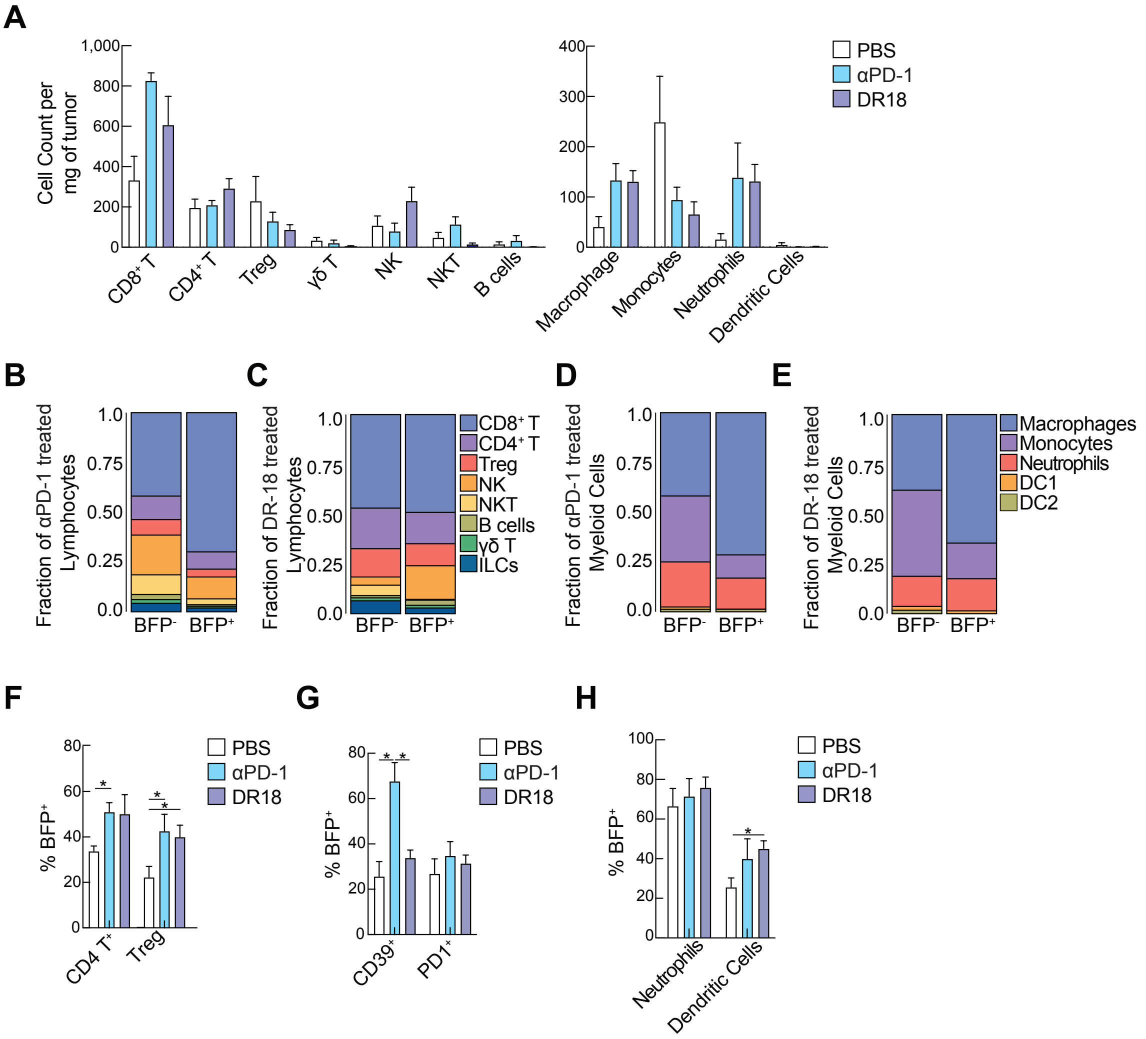

Figure S4. Related to Figure 4&5. Tumor-Immune interactomes following immunotherapy. TR mice were generated and subsequently challenged with YMR senders s.c. On day 7,10 and 13 mice were treated with either vehicle, DR-18, or αPD-1. 14 post injection, intratumoral immune cells were analyzed by flow cytometry. **(a)** Quantification of absolute cell counts in indicated immune cell populations. **(b,c)** Proportion of indicated lymphocytes cell population following αPD-1 **(b)** and DR-18 **(c)** treatment assessed by absolute cell number of BFP^+ or BFP^- cells. **(d,e)** Proportion of indicated myeloid cell population following αPD-1 **(d)** and DR-18 **(d)** treatment assessed by absolute cell number of BFP^+ or BFP^- cells. **(f-h)** Quantification of BFP expression in **(f)** indicated lymphocyte cell population, **(g)** indicated subset of $CD4^+$ T cells, and **(h)** indicated myeloid population. Data is representative of at least n=3 mice per group.
